# Supplementary material for: Genomic language model predicts protein co-regulation and function
Source: Nat Commun. 2024 Apr 3;15:2880. doi: 10.1038/s41467-024-46947-9 (PMC10991518; doi:10.1038/s41467-024-46947-9)
Supplement: Supplementary file 1 — Supplementary Information [file 41467_2024_46947_MOESM1_ESM.pdf]

Supplementary Information for:
Genomic language model predicts protein co-regulation and function

Yunha Hwang[1*], Andre L. Cornman[2], Elizabeth H. Kellogg[3 †], Sergey Ovchinnikov[4*‡], Peter R. Girguis[1*]

[1] Department of Organismic and Evolutionary Biology, Harvard University, Cambridge, MA, USA
[2] Tatta Bio, Baltimore, MD, USA
[3] Department of Molecular Biology and Genetics, Cornell University, Ithaca, NY, USA
[4] John Harvard Distinguished Science Fellowship Program, Harvard University, Cambridge, MA, USA

* co-correspondence: yhwang@g.harvard.edu, so3@mit.edu, pgirguis@oeb.harvard.edu
† Current affiliation: Department of Structural Biology, St. Jude Children's Research Hospital, Memphis TN, USA
‡ Current affiliation: Department of Biology, Massachusetts Institute of Technology, Cambridge, MA, USA

Supplementary Figures:

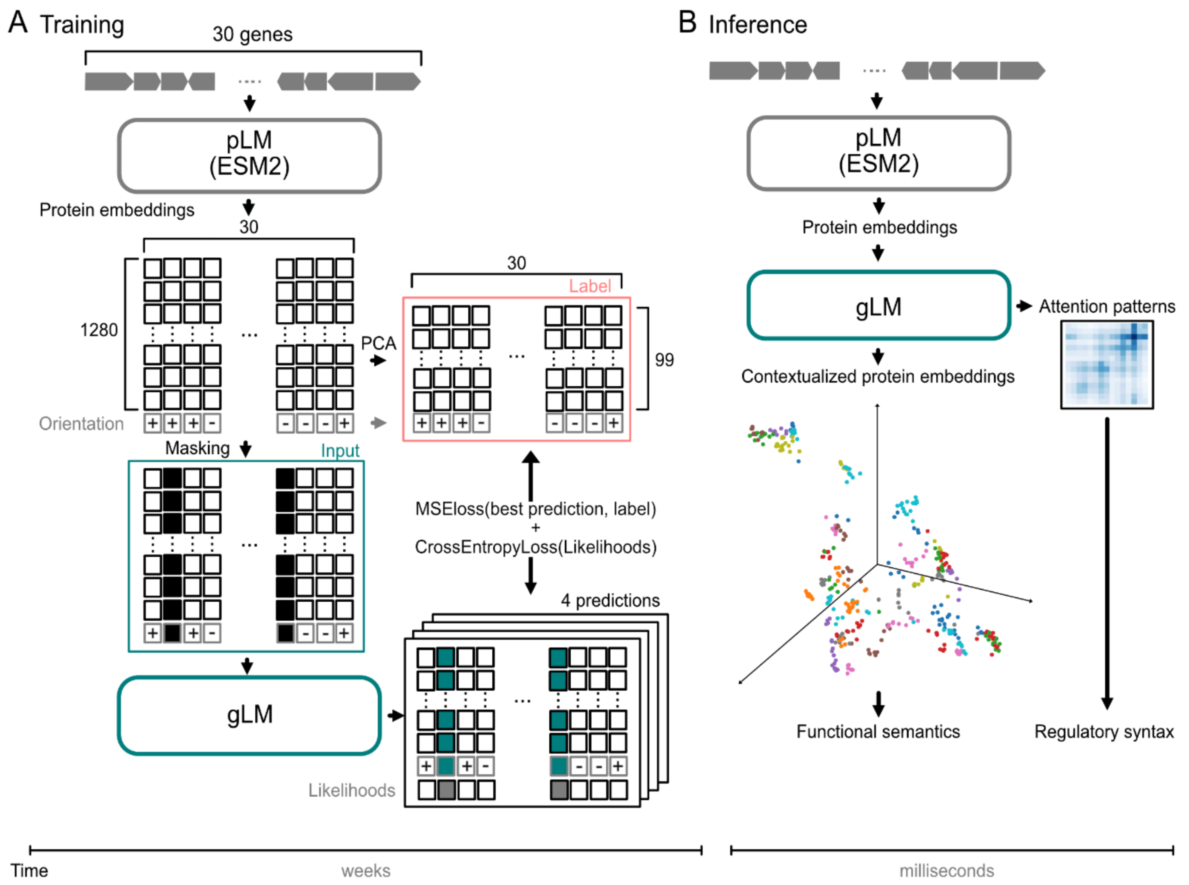

Supplementary Figure 1. Detailed gLM training and inference schematics. A) Training begins with converting 15-30 gene metagenomic subcontigs to protein embeddings using ESM2. Orientation feature is concatenated for each protein and 15% of the proteins are masked randomly to generate training inputs. Labels are generated by applying PCA dimensionality reduction on the ESM2 protein embeddings, and concatenating the orientation feature. gLM is trained to make four possible predictions for the masked tokens, and their associated likelihoods. Training loss is calculated on both the prediction and likelihoods. The training stage takes several weeks on four NVIDIA A100 GPUs. **B)** At inference time, inputs are generated from a metagenomic subcontig using ESM2 output concatenated with an orientation feature. Hidden states and attention patterns of the trained gLM can be used for various downstream tasks.

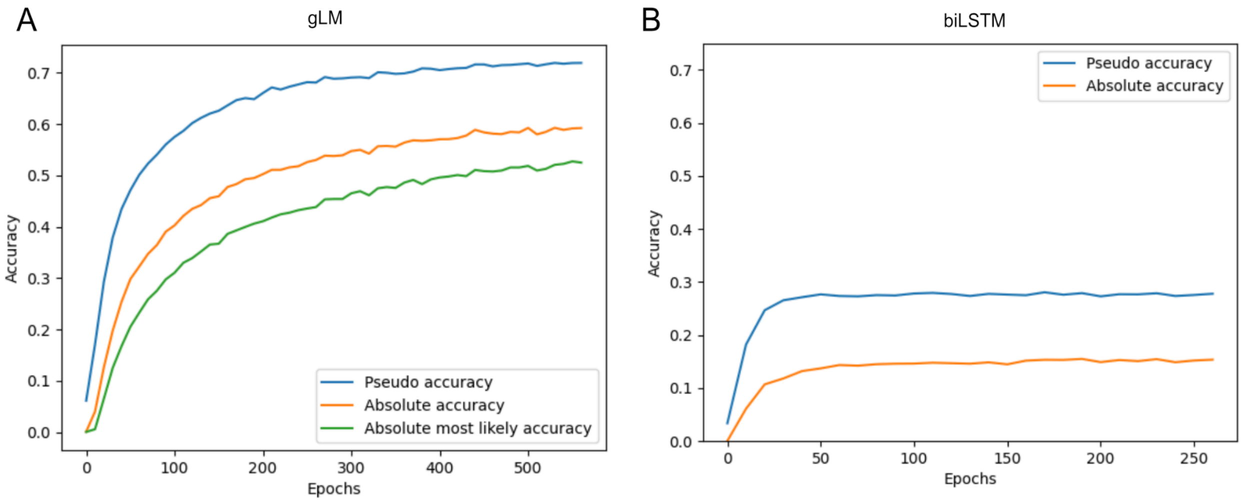

Supplementary Figure 2. Validation accuracy curves for gLM (A) and biLSTM baseline (B). .
Source data are provided as a Source Data file.

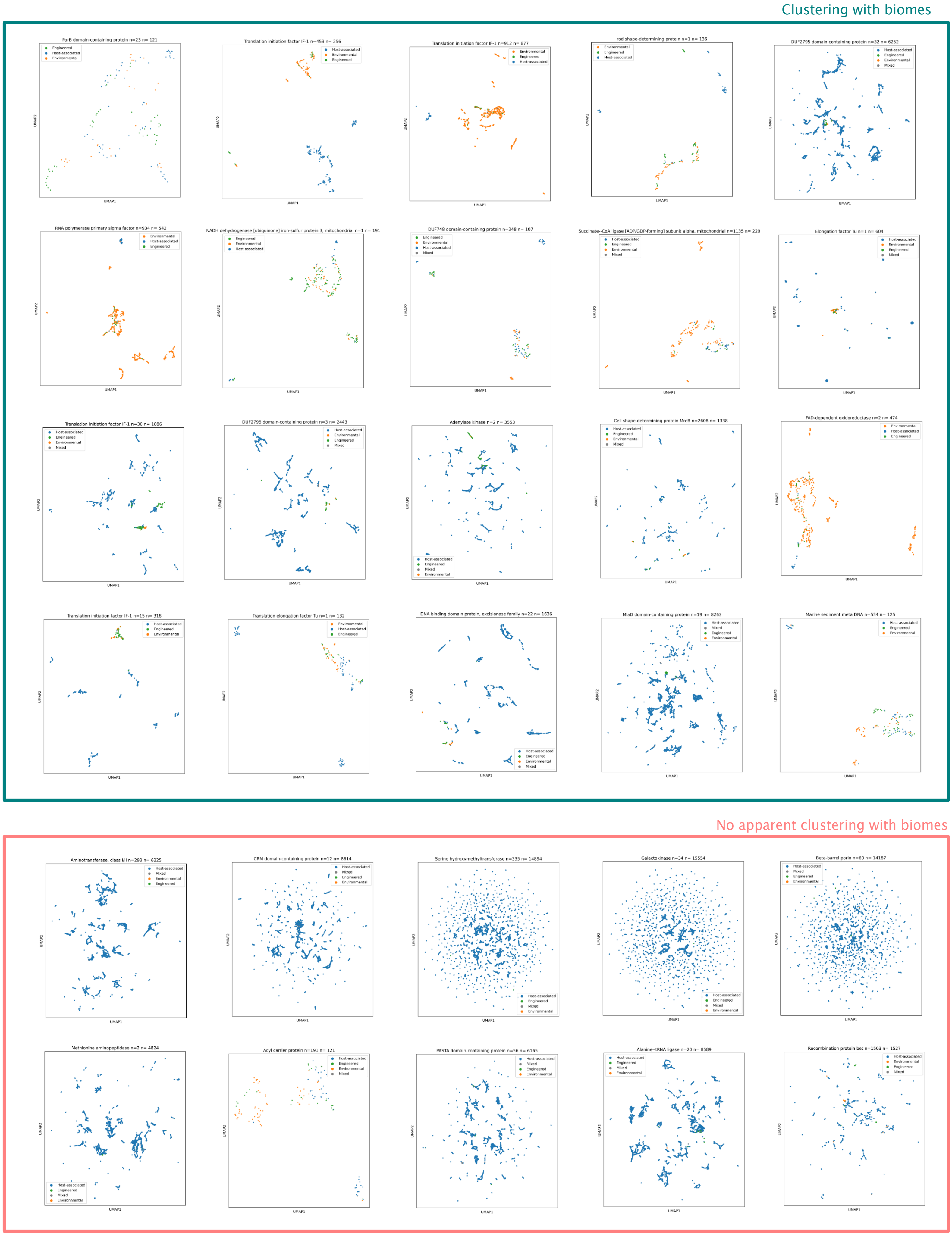
Clustering with biomes
ParB domain-containing protein n=23 n= 121
Translation initiation factor IF-1 n=453 n= 256
Translation initiation factor IF-1 n=912 n= 877
rod shape-determining protein n=1 n= 136
DUF2795 domain-containing protein n=32 n= 6252
RNA polymerase primary sigma factor n=934 n= 542
NADH dehydrogenase [ubiquinone] iron-sulfur protein 3, mitochondrial n=1 n= 191
DUF748 domain-containing protein n=248 n= 107
Succinate--CoA ligase [ADP/GDP-forming] subunit alpha, mitochondrial n=1135 n= 229
Elongation factor Tu n=1 n= 604
Translation initiation factor IF-1 n=30 n= 1886
DUF2795 domain-containing protein n=3 n= 2443
Adenylate kinase n=2 n= 3553
Cell shape-determining protein MreB n=2608 n= 1338
FAD-dependent oxidoreductase n=2 n= 474
Translation initiation factor IF-1 n=15 n= 318
Translation elongation factor Tu n=1 n= 132
DNA binding domain protein, excisionase family n=22 n= 1636
MlaD domain-containing protein n=19 n= 8263
Marine sediment meta DNA n=534 n= 125
No apparent clustering with biomes
Aminotransferase, class I/II n=293 n= 6225
CRM domain-containing protein n=12 n= 8614
Serine hydroxymethyltransferase n=335 n= 14894
Galactokinase n=34 n= 15554
Beta-barrel porin n=60 n= 14187
Methionine aminopeptidase n=2 n= 4824
Acyl carrier protein n=191 n= 121
PASTA domain-containing protein n=56 n= 6165
Alanine--tRNA ligase n=20 n= 8589
Recombination protein bet n=1503 n= 1527
Host-associated
Environmental
Engineered
Mixed
UMAP1
UMAP2

Supplementary Figure 3. Contextualized protein embeddings for all 30 other multi-biome MGYPs. Biome color legend is identical to **Figure 2B**. Blue: Host-associated, Green: Engineered, Orange: Environmental, Grey: Mixed. Source data are provided as a Source Data file.

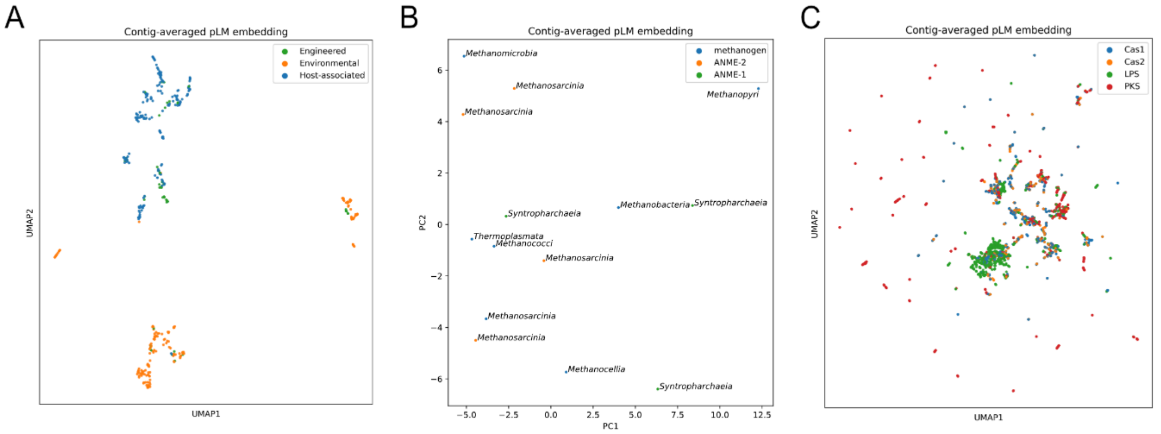

Supplementary Figure 4. Contig-averaged pLM embeddings for figures 2B, F, and I respectively. Contig-averaged pLM embeddings were calculated by mean-pooling pLM embeddings across a given subcontig. Silhouette scores for clustering based on biomes (A), preferred direction of enzymatic reaction (B) and functional category (C) are 0.08, 0.067, and 0.05±0.17 (n = 10 independent samples) respectively. Source data are provided as a Source Data file.

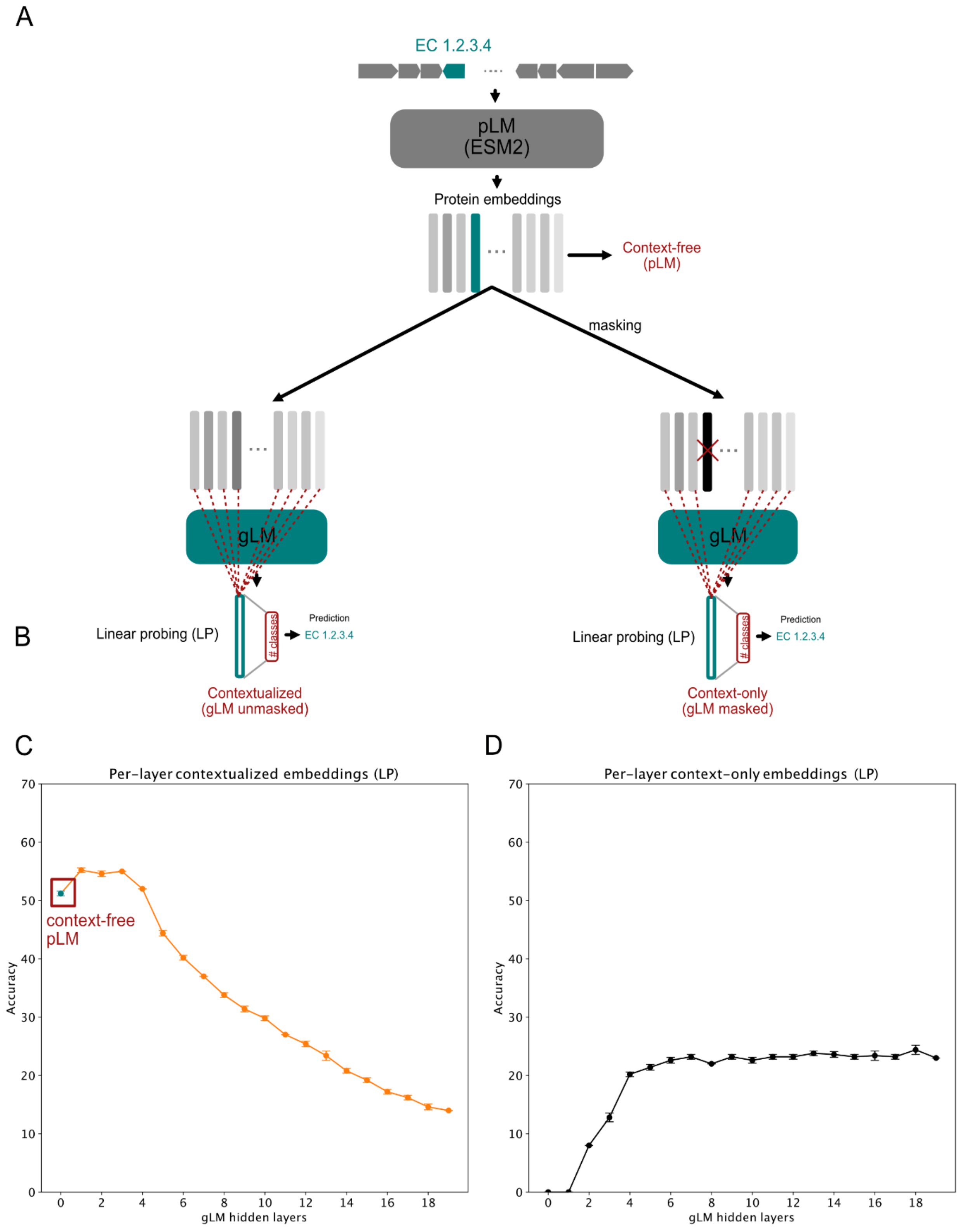
A
EC 1.2.3.4
pLM
(ESM2)
Protein embeddings
Context-free
(pLM)
masking
gLM
gLM
B
Linear probing (LP)
classes
Prediction
EC 1.2.3.4
Contextualized
(gLM unmasked)
Linear probing (LP)
classes
Prediction
EC 1.2.3.4
Context-only
(gLM masked)
C
Per-layer contextualized embeddings (LP)
context-free
pLM
Accuracy
gLM hidden layers
D
Per-layer context-only embeddings (LP)
Accuracy
gLM hidden layers

Supplementary Figure 5. Linear probing of context-free, context-only and contextualized gene embeddings.

A) Schematics of how context-free, context-only, and contextualized gene embeddings are extracted for linear probing. Context-only gene embeddings are extracted by masking the queried gene, therefore the original pLM signal cannot propagate through to gLM representation. This can be used to quantify how gLM-learned context informs EC number prediction independent of pLM signal. B) Schematics of how LP is used for EC number classification. C) Per-layer linear probing accuracy of gLM contextualized embeddings, where no-masking was performed at the time of inference. The 0th layer of gLM is equivalent to context-free pLM embedding. D) Per-layer linear probing accuracies of gLM context-only embeddings, where the queried genes are masked at the time of inference. Source data are provided as a Source Data file.

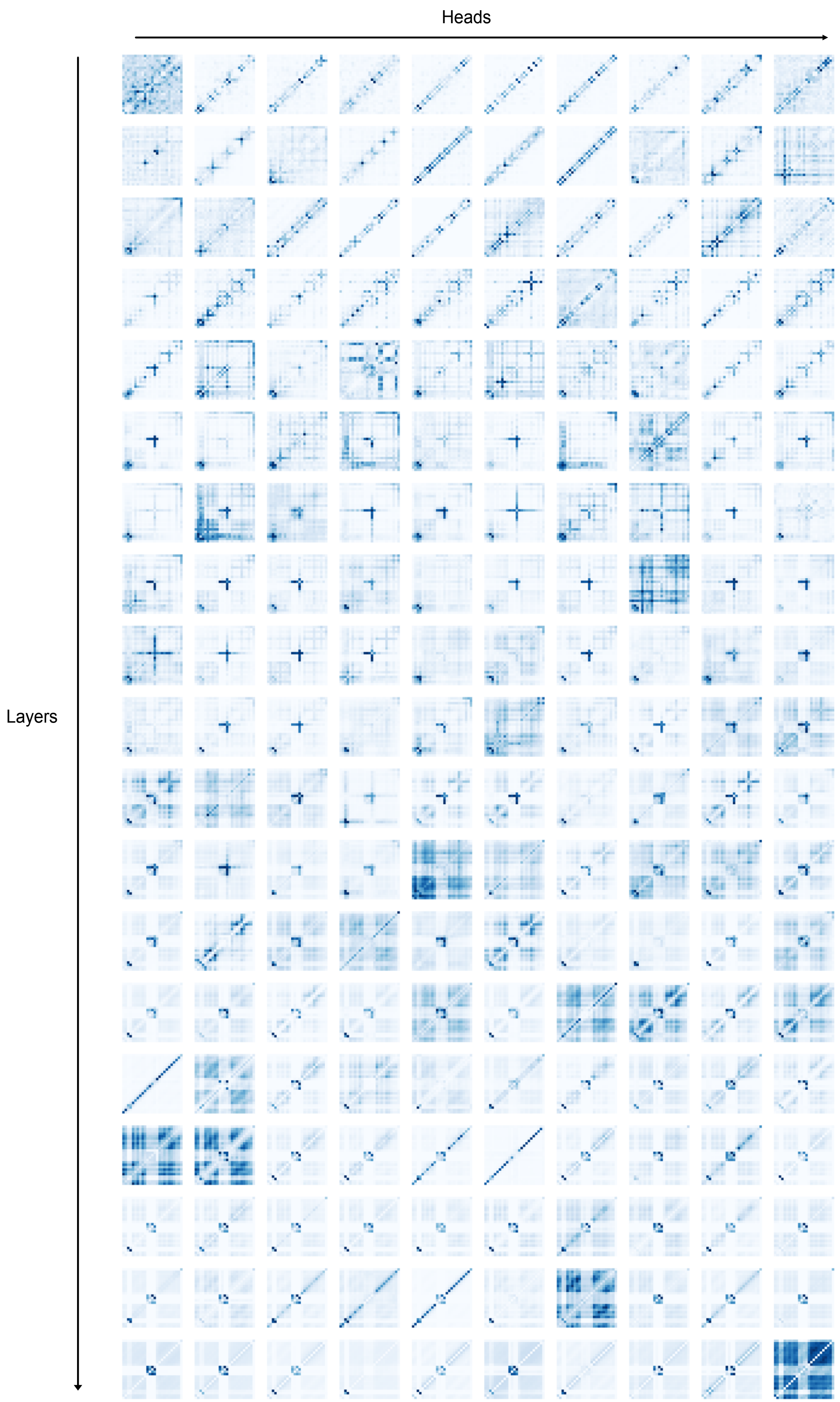
Heads
Layers

Supplementary Figure 6. Visualization of attention heads for a randomly chosen sequence. Increasing layer depth down the figure. Darker color represents larger attention scores.

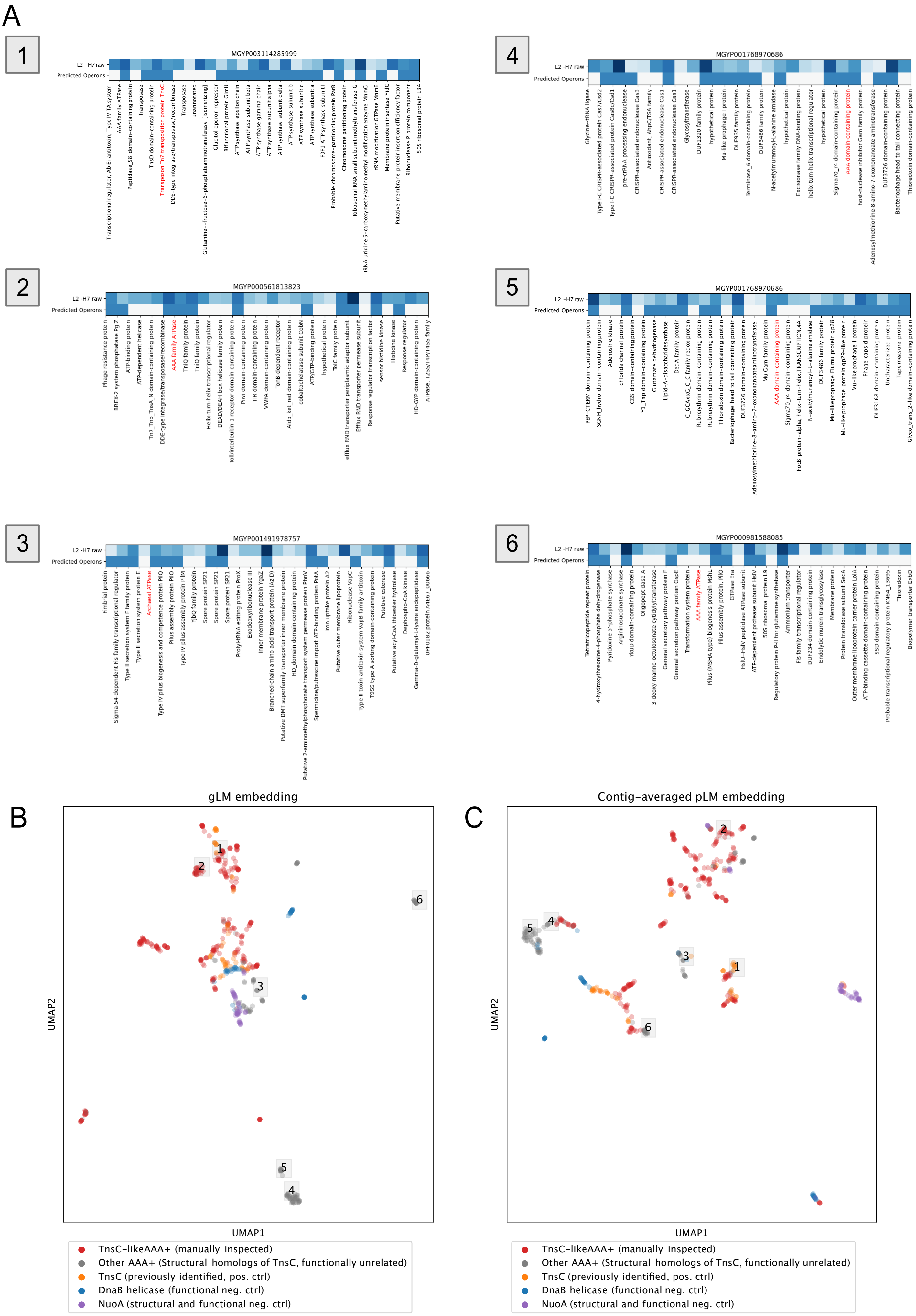
A
MGYP003114285999
MGYP000561813823
MGYP001491978757
MGYP001768970686
MGYP001768970686
MGYP000981588085
L2 -H7 raw
Predicted Operons
B
gLM embedding
C
Contig-averaged pLM embedding
UMAP1
UMAP2
TnsC-likeAAA+ (manually inspected)
Other AAA+ (Structural homologs of TnsC, functionally unrelated)
TnsC (previously identified, pos. ctrl)
DnaB helicase (functional neg. ctrl)
NuoA (structural and functional neg. ctrl)

Supplementary Figure 7. Attention-based operon prediction of AAA+ regulators. A) Annotations were made by searching against the UniRef90 database. Protein annotation in Red indicates the location of the AAA+ regulator identified based on a structural search of TsnC. Raw attention in the most correlated head (2nd layer, 7nd head, "L2-H7") is shown in the first row of the heatmap, and the second row is the predicted operon using the logistic regression predictor trained on *E.coli* K-12 operon dataset. **B)** gLM representations (last hidden layer of gLM) for **Figure 4EF** (KL divergence ratio = 0.53). **C)** Contig-averaged pLM baseline for **Figure 4EF**, where pLM embeddings across the subcontig were averaged to represent the queried protein (KL divergence ratio = 0.57) Source data are provided as a Source Data file.

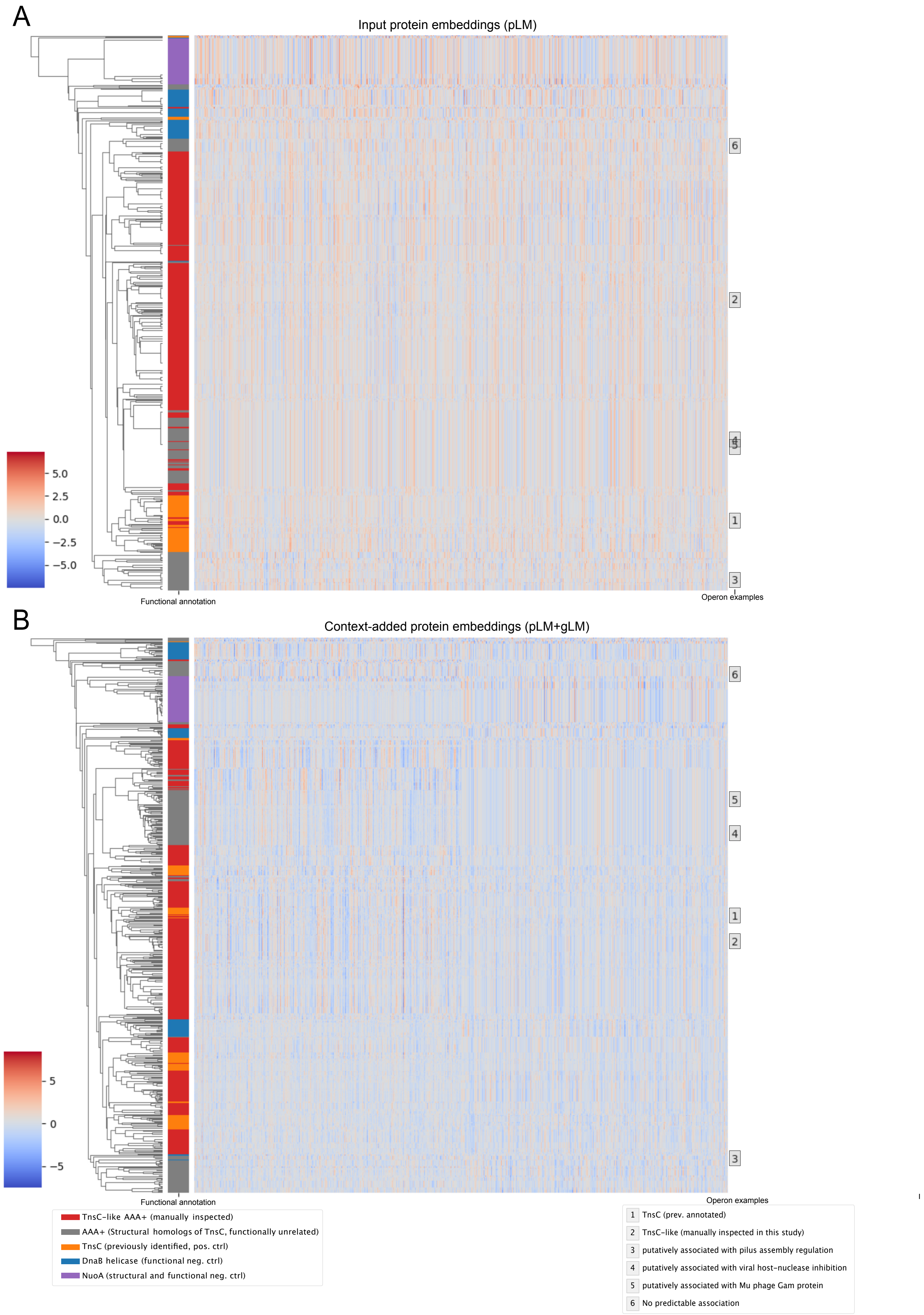
A
Input protein embeddings (pLM)
5.0
2.5
0.0
−2.5
−5.0
Functional annotation
Operon examples
6
2
4
5
1
3
B
Context-added protein embeddings (pLM+gLM)
5
0
−5
Functional annotation
Operon examples
6
5
4
1
2
3
TnsC-like AAA+ (manually inspected)
AAA+ (Structural homologs of TnsC, functionally unrelated)
TnsC (previously identified, pos. ctrl)
DnaB helicase (functional neg. ctrl)
NuoA (structural and functional neg. ctrl)
1 TnsC (prev. annotated)
2 TnsC-like (manually inspected in this study)
3 putatively associated with pilus assembly regulation
4 putatively associated with viral host-nuclease inhibition
5 putatively associated with Mu phage Gam protein
6 No predictable association

Supplementary Figure 8. Distance based clustering of raw embeddings of AAA+ regulators and controls shown in Figure 4EF. Row colors and labels correspond to the point colors and designation used in **Figure 4EF** and **Supplementary Figure 7**. Source data are provided as a Source Data file.

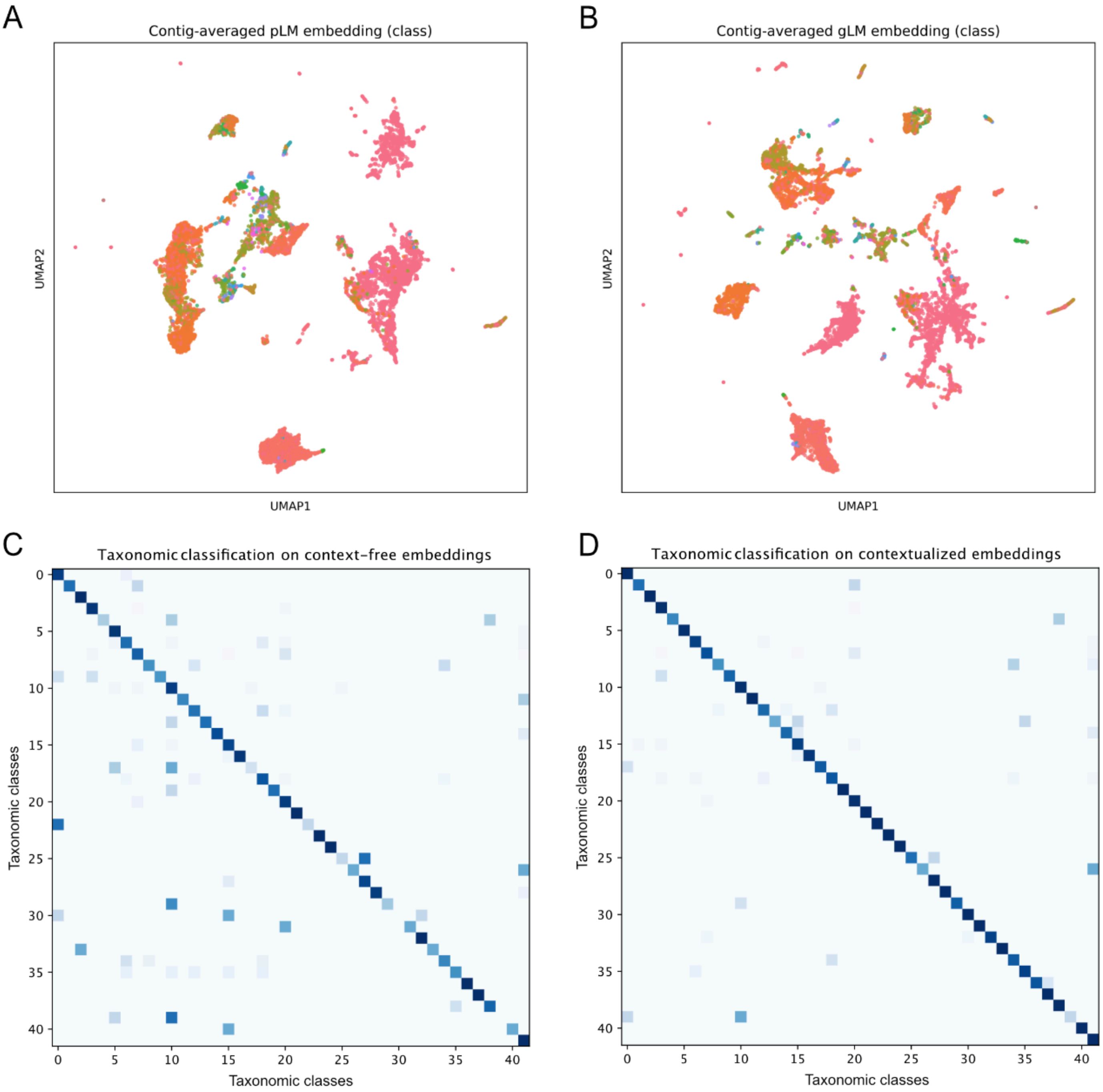

Supplementary Figure 9. Contextualization of contig embeddings and taxonomic classification. Comparison of context-free (A) and contextualized (B) class-level taxonomy of 30-gene contigs as shown in **Figure 5G**. Confusion matrices for context-free (C) and contextualized (D) logistic regression classifier as shown in **Figure 5F**. Source data are provided as a Source Data file.

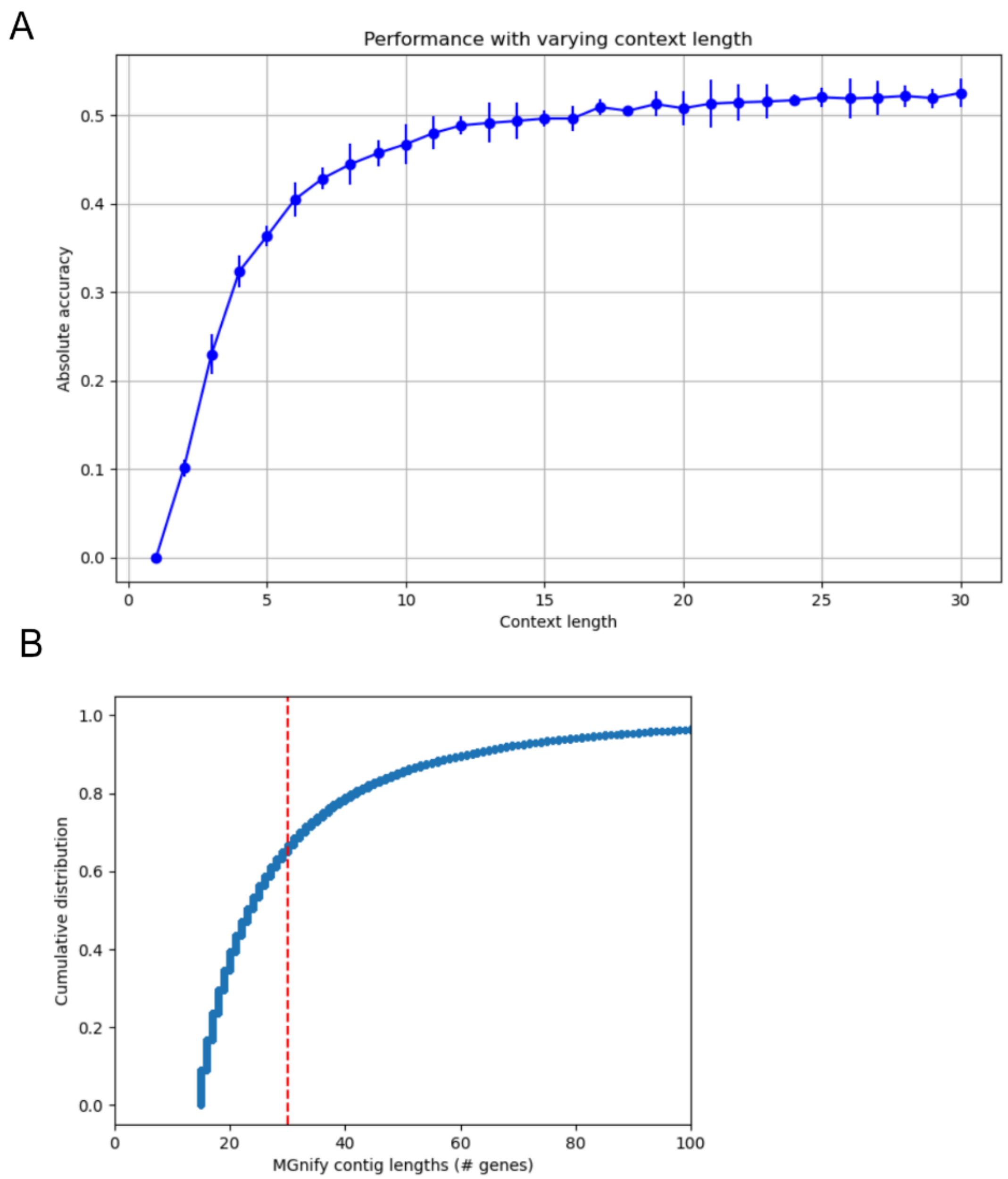

Supplementary Figure 10. A) Increasing model performance (estimated by absolute accuracy on *E. coli* K-12) with increasing context length (1 to 30 genes). See Methods section "Performance metric and validation" for detailed absolute accuracy calculation. Five random sets of genes in E. coli K-12 genome (# genes = 4315) were used for error estimation. Data are presented as mean values +/- standard deviation over five random subsegments of the *E. coli* K-12 genome. B). Cumulative distribution of raw MGnify contig lengths (count of encoded genes), MGnify contigs < 15 genes were excluded from the corpus in this study. Red line is drawn at the contig length of 30 genes. Source data are provided as a Source Data file.

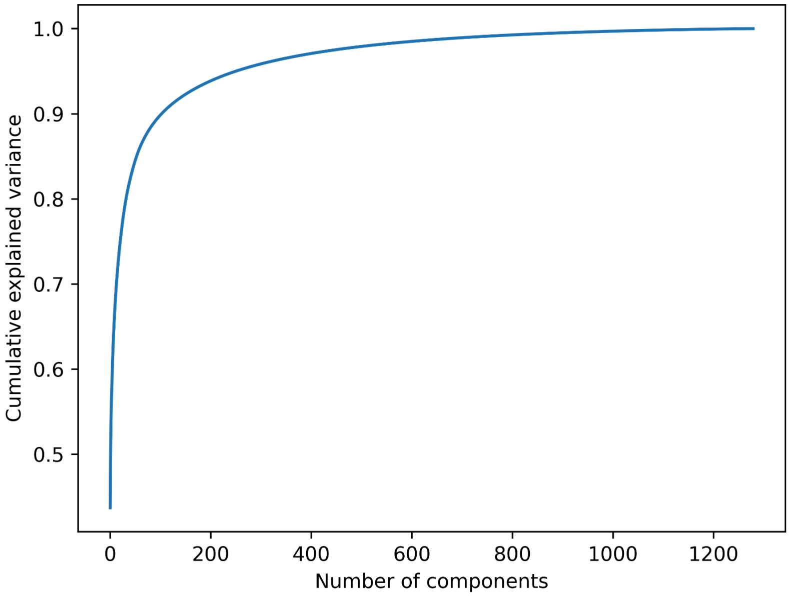

Supplementary Figure 11. Cumulative explained variance of principal components of ESM2 embeddings calculated on 2.5 million randomly selected MGYPs. Source data are provided as a Source Data file.

Supplementary Tables:

Supplementary Table 1. Comparison of gLM to previous efforts in modeling various aspects of biological sequences.

	**Multi-gene input**	**Continuous representation of genes**	**Generalizable across organisms (Organism-agnostic pretraining)**	**Self-supervised language model**
gLM (this study)	✓ (Max context of 30 genes)	✓ (Model inputs and outputs are continuous gene representations)	✓ (Metagenomic sequences with bias towards bacteria, archaea and viruses)	✓ (Masked language model)
pLMs[1–4](e.g. ESM2, ProtBert, ProtT5, ProGen, CaLM)	✕ (Max context of 1 gene)	✓ (Continuous gene representation can be extracted from pooled intermediate layer)	✓ (ESM2 Trained on Uniref-50)	✓ (Masked language model)
Miller et al[5]	✓ (Trained with max context of 5 genes)	✕ (Discrete gene vocab size of 563,589)	✓ (Trained on NCBI WGS and EBI MGnify, excluding green plants, fungi, and animals)	✕ (Based on word2vec, a one-layer word embedding model, not a language model)
Enformer[6]	✓ (Max context 200 kbp)	✓ (Continuous gene representation can be extracted from pooled intermediate layer)	✕ (Pretrained on human and mouse genomes only)	✕ (Supervised with 5,313 human genomic tracks and 1,643 mouse genomic tracks)
DNABERT[7]	✕ (Max context length of DNABERT-6 is 3072 bp, which is not sufficient to include a median length (26,288 bp) human protein coding gene[8])	✓ (Continuous gene representation can be extracted from pooled intermediate layer)	✕ (Pretrained on human genome)	✓ (Masked language model)
Nucleotide Transformer[9]	✕ (Max context of 6000 bp, which is not sufficient to include a median length (26,288 bp) human protein coding gene)	✓ (Continuous gene representation can be extracted from pooled intermediate layer)	✕ (Heavily biased towards human genome)	✓ (Masked language model)
HyenaDNA[10]	✓ (Max context of 1M bp)	✓ (Continuous gene representation can be extracted from pooled intermediate layer)	✕ (Pretrained on Human genome)	✓ (Causal language model)
GenSLM-foundation model[11]	✕ (Max context of 1 gene during pretraining)	✓ (Continuous gene representation can be extracted from pooled intermediate layer)	✓ (Pretrained on BV-BRC)	✓ (Causal language model)
GenSLM-SARS-CoV2 genome model[11]	✓ (Max context of 10,240 codons)	✓ (Continuous gene representation can be extracted from pooled intermediate layer)	✕ (Fine-tuned on SARS-CoV2 genomes only)	✓ (Causal language model)

Supplementary Table 2. Comparison of biLSTM baseline model with transformer-based gLM architecture and validation performances. Note that the biLSTM baseline is smaller in model size than gLM. While we attempted to scale this model by increasing the number of layers, the model failed to converge.

	**biLSTM**	**gLM**
Number of layers	5	19
Attention heads	N/A	10
Input embedding dimension	1281	1281
Hidden size	1280	1280
Batch size	4000	3000
Learning rate	1e-4	1e-4
Warm up steps	5000	5000
Training steps	467,253	1,296,960
Number of predictions	1	4
Number of parameters	27,811,840	954,736,916
% Pseudo-accuracy (validation)	27.9	71.9
% Absolute accuracy (validation)	14.78	59.2

Supplementary Table 3. Ablation of pLM representations. Ablated gLM was trained on one-hot representations until convergence (<0.1% decrease in loss over 40k iterations).

	**gLM**	**gLM one-hot**
Representations	ESM2 embedding	One-hot amino acid encoding
Pooling	mean	mean
Number of layers	19	19
Attention heads	10	10
Input embedding dimension	1281	34
Hidden size	1280	1280
Batch size	3000	3000
Learning rate	1e-4	1e-4
Warm up steps	5000	5000
Training steps	1296960	132050
Number of predictions	4	4
Number of parameters	954736916	945338764
% Pseudo-accuracy (validation)	71.9	3.29
% Absolute accuracy (validation)	59.2	0.002
Operon prediction mAP (± SD)	0.775 ± 0.028	0.426 ± 0.015

Supplementary Table 4. Context-variant gene annotations.

A. List of top ten most context variant genes where variance is calculated using gLM contextualized embeddings.

MGYP ID	Mean embedding variance	UniRef annotation
815530568	18.87014775	Uncharacterized protein n=1
3385096772	17.70851124	Tail fiber protein n=20
2546832411	17.70269541	Capsular exopolysaccharide family n=1
767058056	17.08193362	Type I restriction enzyme endonuclease subunit
3381572716	16.44215407	Ferrous iron transport protein B n=11
2534762610	16.38703203	None
2733868861	16.21466745	30S ribosomal protein S18 n=4
551790786	16.06301055	30S ribosomal protein S18 n=285
3383952544	15.74879686	CRISPR-associated endoribonuclease Cas2 n=88
832772145	15.62986097	Ferrous iron transport protein A n=6

B. List of top most context variant genes where variance is calculated using contig-Averaged.

MGYP ID	Mean embedding variance	UniRef annotation
3384243084	18.43362238	DDE_Tnp_1 domain-containing protein n=137
943740416	17.77505976	Phosphoribosylamine--glycine ligase n=7
120256991	16.91703339	Asparaginase n=7
209907419	16.75686986	DNA mismatch repair protein MutS n=2
3380534673	16.67205026	IS66-like element ISBf10 family transposase
3384295730	16.44076785	IS66-like element ISBf10 family transposase
3381576767	16.23692972	VOC domain-containing protein n=96
3385319961	16.10454523	Peptidase E n=538
87924424	15.97918443	3-oxoacyl-[acyl-carrier-protein] synthase 3
997418675	15.91416004	HNH homing endonuclease n=2

Supplementary References

1. Rives, A. *et al.* Biological structure and function emerge from scaling unsupervised learning to 250 million protein sequences. *Proc. Natl. Acad. Sci. U. S. A.* **118**, (2021).
2. Elnaggar, A. *et al.* ProtTrans: Towards Cracking the Language of Life's Code Through Self-Supervised Deep Learning and High Performance Computing. *arXiv [cs.LG]* (2020).
3. Madani, A. *et al.* Large language models generate functional protein sequences across diverse families. *Nat. Biotechnol.* 1–8 (2023).
4. Outeiral, C. & Deane, C. Codon language embeddings provide strong signals for protein engineering. *BioRxiv* (12/2022) doi:10.1101/2022.12.15.519894.
5. Miller, D., Stern, A. & Burstein, D. Deciphering microbial gene function using natural language processing. *Nat. Commun.* **13**, 5731 (2022).
6. Avsec, Ž. *et al.* Effective gene expression prediction from sequence by integrating long-range interactions. *Nat. Methods* **18**, 1196–1203 (2021).
7. Ji, Y., Zhou, Z., Liu, H. & Davuluri, R. V. DNABERT: pre-trained Bidirectional Encoder Representations from Transformers model for DNA-language in genome. *Bioinformatics* **37**, 2112–2120 (2021).
8. Piovesan, A., Caracausi, M., Antonaros, F., Pelleri, M. C. & Vitale, L. GeneBase 1.1: a tool to summarize data from NCBI gene datasets and its application to an update of human gene statistics. *Database* **2016**, (2016).
9. Dalla-Torre, H. *et al.* The Nucleotide Transformer: Building and Evaluating Robust Foundation Models for Human Genomics. *bioRxiv* 2023.01.11.523679 (2023) doi:10.1101/2023.01.11.523679.
10. Nguyen, E. *et al.* HyenaDNA: Long-Range Genomic Sequence Modeling at Single Nucleotide Resolution. (2023).
11. Zvyagin, M. *et al.* GenSLMs: Genome-scale language models reveal SARS-CoV-2 evolutionary dynamics. *bioRxiv* (2022) doi:10.1101/2022.10.10.511571.
