# Supplementary material for: Genomic language model predicts protein co-regulation and function
Source: Nat Commun. 2024 Apr 3;15:2880. doi: 10.1038/s41467-024-46947-9 (PMC10991518; doi:10.1038/s41467-024-46947-9)
Supplement: Supplementary file 3 — Reporting Summary [file 41467_2024_46947_MOESM3_ESM.pdf]

nature portfolio

Corresponding author(s): Yunha Hwang, Sergey Ovchinnikov, Peter Girguis

Last updated by author(s): Jan 17, 2024

Reporting Summary

Nature Portfolio wishes to improve the reproducibility of the work that we publish. This form provides structure for consistency and transparency in reporting. For further information on Nature Portfolio policies, see our Editorial Policies and the Editorial Policy Checklist.

Statistics

For all statistical analyses, confirm that the following items are present in the figure legend, table legend, main text, or Methods section.

n/a	Confirmed	
☐	☒	The exact sample size (*n*) for each experimental group/condition, given as a discrete number and unit of measurement
☐	☒	A statement on whether measurements were taken from distinct samples or whether the same sample was measured repeatedly
☐	☒	The statistical test(s) used AND whether they are one- or two-sided<br>*Only common tests should be described solely by name; describe more complex techniques in the Methods section.*
☐	☒	A description of all covariates tested
☐	☒	A description of any assumptions or corrections, such as tests of normality and adjustment for multiple comparisons
☐	☒	A full description of the statistical parameters including central tendency (e.g. means) or other basic estimates (e.g. regression coefficient) AND variation (e.g. standard deviation) or associated estimates of uncertainty (e.g. confidence intervals)
☐	☒	For null hypothesis testing, the test statistic (e.g. *F*, *t*, *r*) with confidence intervals, effect sizes, degrees of freedom and *P* value noted<br>*Give P values as exact values whenever suitable.*
☒	☐	For Bayesian analysis, information on the choice of priors and Markov chain Monte Carlo settings
☐	☒	For hierarchical and complex designs, identification of the appropriate level for tests and full reporting of outcomes
☐	☒	Estimates of effect sizes (e.g. Cohen's *d*, Pearson's *r*), indicating how they were calculated

Our web collection on statistics for biologists contains articles on many of the points above.

Software and code

Policy information about availability of computer code

Data collection	No software was used for data collection.
Data analysis	ESM2 (https://github.com/facebookresearch/esm, "esm2_t33_650M_UR50D" model), huggingface's transformers v4.22.2, python v3.10.8, fair-esm v1.0.2, scikit-learn v1.1.2, scipy v1.9.2, numpy v1.23.3, matplotlib v3.6.2, umap-learn v0.5.3, seaborn v0.12.1, sklearn v0.0, Diamond v2.0.7.145, CD-HIT v4.6

For manuscripts utilizing custom algorithms or software that are central to the research but not yet described in published literature, software must be made available to editors and reviewers. We strongly encourage code deposition in a community repository (e.g. GitHub). See the Nature Portfolio guidelines for submitting code & software for further information.

Data

Policy information about availability of data

All manuscripts must include a data availability statement. This statement should provide the following information, where applicable:

- Accession codes, unique identifiers, or web links for publicly available datasets
- A description of any restrictions on data availability
- For clinical datasets or third party data, please ensure that the statement adheres to our policy

Dataset used for training is available for download from the MGnify server (http://ftp.ebi.ac.uk/pub/databases/metagenomics/peptide_database/2022_05/).

Dataset used for annotation is UniRef90 (https://www.uniprot.org/help/downloads). The model is available on Zenodo under accession number 10.5281/zenodo.7855545.

Research involving human participants, their data, or biological material

Policy information about studies with human participants or human data. See also policy information about sex, gender (identity/presentation), and sexual orientation and race, ethnicity and racism.

Reporting on sex and gender	N/A
Reporting on race, ethnicity, or other socially relevant groupings	N/A
Population characteristics	N/A
Recruitment	N/A
Ethics oversight	N/A

Note that full information on the approval of the study protocol must also be provided in the manuscript.

Field-specific reporting

Please select the one below that is the best fit for your research. If you are not sure, read the appropriate sections before making your selection.

☐ Life sciences ☐ Behavioural & social sciences ☒ Ecological, evolutionary & environmental sciences

For a reference copy of the document with all sections, see nature.com/documents/nr-reporting-summary-flat.pdf

Ecological, evolutionary & environmental sciences study design

All studies must disclose on these points even when the disclosure is negative.

Study description	Design and analysis of unsupervised genomic language model trained on large metagenomic sequence datasets.
Research sample	Metagenome sequence data. Metagenomic datasets were used for training the model, such that the model learns from the most diverse and least biased genomic data. The metagenomic samples are drawn from microbial communities across biomes (e.g. ocean, soil, host-associated)
Sampling strategy	We sampled the MGnify dataset to include all metagenomic contigs that encoded greater than 15 genes. This threshold was chosen to ensure that each genomic sequence sample contained sufficient genomic context information, such that the model can be trained with computational efficiency (less padding).
Data collection	Data was collected from EMBL-EBI's MGnify server by Yunha Hwang
Timing and spatial scale	Data was downloaded from the server (http://ftp.ebi.ac.uk/pub/databases/metagenomics/peptide_database/2022_05/) on 15 June 2022. Download was conducted once.
Data exclusions	~1% of the samples contained proteins that were not linked to MGnify's representative protein clusters due to minor issues in the dataset build. These samples were excluded from our dataset after consultation with the MGnify team.
Reproducibility	Model training was replicated by running the training script on smaller subsets of the training data and observing similar learning dynamics. The final model was not replicated due to high computational cost of training a full-scale model. All attempts to repeat downstream experiments (by re-running the analysis scripts on a different random subset of the analysis data) were successful.
Randomization	This is not relevant for this study because we used the whole dataset without any supervision (no labels were assigned) for training.
Blinding	Blinding is not relevant for this study because we are modeling genomic sequences without any explicit labels.

Did the study involve field work? ☐ Yes ☒ No

Reporting for specific materials, systems and methods

We require information from authors about some types of materials, experimental systems and methods used in many studies. Here, indicate whether each material, system or method listed is relevant to your study. If you are not sure if a list item applies to your research, read the appropriate section before selecting a response.

Materials & experimental systems

n/a	Involved in the study
☒	☐ Antibodies
☒	☐ Eukaryotic cell lines
☒	☐ Palaeontology and archaeology
☒	☐ Animals and other organisms
☒	☐ Clinical data
☒	☐ Dual use research of concern
☒	☐ Plants

Methods

n/a	Involved in the study
☒	☐ ChIP-seq
☒	☐ Flow cytometry
☒	☐ MRI-based neuroimaging
